# Reducing human pressure on farmland could rescue China’s declining wintering geese

**DOI:** 10.1186/s40462-020-00220-y

**Published:** 2020-08-18

**Authors:** Yali Si, Jie Wei, Wenzhao Wu, Wenyuan Zhang, Lin Hou, Le Yu, Ben Wielstra

**Affiliations:** 1 Ministry of Education Key Laboratory for Earth System Modeling, Department of Earth System Science, Tsinghua University, Beijing, China; 2 Institute of Environmental Sciences, Leiden University, Leiden, Netherlands; 3 Centre for Healthy Cities, Institute for China Sustainable Urbanization, Tsinghua University, Beijing, China; 4 Department of Zoology, University of Oxford, Oxford, UK; 5 Center for Statistical Science, Tsinghua University, Beijing, China; 6 Institute of Biology Leiden, Leiden University, Leiden, The Netherlands; 7 Naturalis Biodiversity Center, Leiden, The Netherlands

**Keywords:** Yangtze River floodplain, Northeast China plain, Satellite tracking, Habitat use, Refuge, Alternative feeding area, Agricultural land, Resource selection function model

## Abstract

**Background:**

While goose populations worldwide benefit from food provided by farmland, China’s threatened wintering goose populations have failed to capitalize on farmland. It has been proposed that, due to an exceptionally intense human pressure on Chinese farmland, geese cannot exploit farmland in their wintering sites and hence are confined to their deteriorating natural habitat. If this were true, locally decreasing this human pressure on farmland ‘refuges’ would represent a promising conservation measure.

**Methods:**

We investigate habitat use of two declining migratory goose species in their core wintering (Yangtze River Floodplain) and stopover (Northeast China Plain) regions, compare the human pressure level at both regions, and adopt a mixed-effect resource selection function model to test how human pressure, food resource type (farmland or wetland/grass), distance to roosts, and their interaction terms influence the utilization of food resources for each species and region. To this aim we use satellite tracking of 28 tundra bean geese *Anser serrirostris* and 55 greater white-fronted geese *A. albifrons*, a newly produced 30 m land cover map, and the terrestrial human footprint map.

**Results:**

Geese use farmland intensively at their stopover site, but hardly at their wintering site, though both regions have farmland available at a similar proportion. The human pressure on both farmland and wetland/grass is significantly lower at the stopover region compared to the wintering region. At both sites, the two goose species actively select for farmland and/or wetland/grass with a relatively low human pressure, positioned relatively close to their roosting sites.

**Conclusions:**

Our findings suggest that if human pressure were to decrease in the farmlands close to the roost, China’s wintering geese could benefit from farmland. We recommend setting aside farmland near roosting sites that already experiences a relatively low human pressure as goose refuges, and adopt measures to further reduce human pressure and increase food quality and quantity, to help counter the decline of China’s wintering goose populations. Our study has important conservation implications and offers a practical measure for migratory waterfowl conservation in areas of high human-wildlife conflict.

## Background

Chinese wintering geese have rapidly declined [1]. While wintering goose populations have benefited from the increased food supply provided by agricultural land across most of the Northern Hemisphere [2, 3], this appears not to be the case in China. It has been proposed that, due to the exceptionally intense human pressure on China’s farmland, the goose populations wintering in China cannot exploit the riches provided by agricultural land and hence geese are ‘imprisoned’ here inside their degrading natural habitat [4]. In general, geese respond negatively to human disturbance as a net loss in energy stores, resulting from a decreased intake and an increased expenditure related to avoidance behavior, which reduces migration and breeding success [5, 6].

The corollary is that, if human pressure on farmland were to decrease, geese in China could take advantage of farmland. This would have important conservation implications because ‘refuges’ – patches of farmland where human pressure is reduced as they are temporarily set aside as alternative feeding area for birds – have shown to boost goose populations in other parts of the world [3, 7–11]. If it can be shown that Chinese wintering geese do venture into farmland in regions where human pressure is lower, this would demonstrate that these geese are likely to benefit from the creation of farmland refuges in their wintering site.

**Fig. 1:**
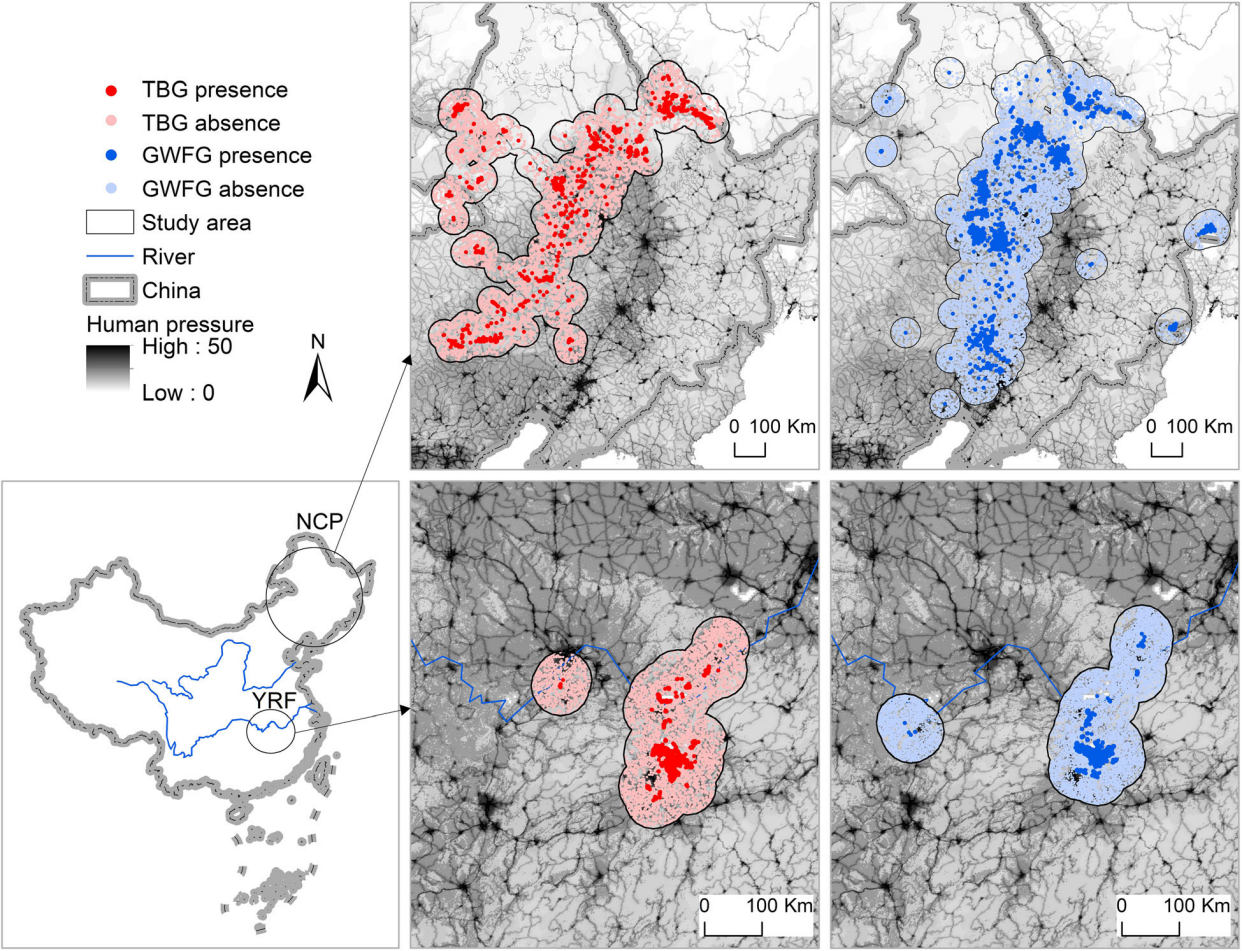
Study area and GPS locations of two goose species at their stopover and wintering sites. The study area is the range of a 50 km-buffer around day GPS locations of each goose species. Human pressure refers to the standardized human footprint with a range of 0–50. NCP: Northeast China Plain (the stopover region); YRF: Yangtze River Floodplain (the wintering region); TBG: tundra bean geese (*Anser serrirostris*); GWFG: greater white-fronted geese (*A. albifrons*). Red dots indicate TBG GPS records (dark red) and generated absences (light red) and blue dots indicate GWFG GPS records (dark blue) and generated absences (light blue)

We test our prediction by taking advantage of the fact that human pressure on farmland shows spatial variation across China [12, 13]. To this aim, we use satellite tracking data (Fig. 1) of tundra bean geese (*Anser serrirostris*; *n* = 28) and greater white-fronted geese (*A. albifrons*; *n* = 55) that winter in the Yangtze River Floodplain and use the Northeast China Plain as a core stopover site, during their migration to and from the Arctic breeding sites [14]. As a proxy for human disturbance on farmland, we adopt the recently published Global Terrestrial Human Footprint Map, a cumulative human footprint measure based on infrastructure, land cover and human access into natural areas [12, 13].

For both goose species we 1) compare the human pressure between the wintering and the stopover region and 2) investigate habitat use at both the wintering and the stopover region. Furthermore, we 3) test whether geese actively select for resources with a relatively low human pressure, by building a mixed-effect resource selection model using human pressure, resource types (farmland and wetland/grass), distance to roosts, and their interaction terms as fixed factors, and year and individual as random factors.

## Materials and methods

### Tracking data

Geese were captured at Poyang Lake in the Yangtze River Floodplain, Jiangxi Province, China (29°N, 116°E) during the 2014-2018 wintering seasons, and equipped with GPS - GSM (Global Positioning System - Global System for Mobile Communications), solar- powered loggers. Further background on capture and deployment methods can be found in Si et al. [14]. Each logger was set to record GPS (Global Position System) positions every 2 h. The collected data used in this study include bird ID, latitude and longitude (degree), time of record, and speed (km/hour). We used tracking data from 28 individuals of tundra bean goose and 55 individuals of greater white-fronted goose for further analyses.

The start and end dates of wintering / spring staging in the Yangtze River Floodplain and stopover in the Northeast China Plain for each individual were defined as the first day it arrived/left the specific site and showing continuous presence/absence. For individuals with multi-year data, the year with the highest amount of GPS records was used. Records with a GPS location error over 30 m and/or speed over 1 km/hour (presumably while in flight instead of foraging) were removed from downstream analyses. The sunrise and sunset time were calculated based on the geographic location of each GPS record using algorithms provided by the National Oceanic & Atmospheric Administration (NOAA) (https://www.esrl.noaa.gov/gmd/grad/solcalc/). Day and night locations were defined as the locations recorded in the periods between 1 h before sunrise to 1 h after sunset, and 1 h after sunset to 1 h before sunrise. For each individual bird, day and night counts of locations, start and end dates at the wintering and stopover region, and logger information are summarized in Additional file 1: Table S1.

### Habitat use at both regions

We produced a 2015 China land cover map at a 30 m resolution according to Liu et al. [15] with an overall accuracy over 91% and used this to compare the habitat use of tracked geese at their wintering and stopover regions. Land cover types were regrouped to farmland, woodland, grassland, water, wetland (i.e., swamp, mud flat and bottomland), built-up land, and bare land (i.e., sand, desert, saline-alkali land and bare land). To compare the use of different food resources, land cover types were regrouped and the percentage of tracked locations during the day and at night on farmland, water/wetland/grass (grassland are included to cover wet meadows), and others (the rest land cover types) was calculated in both the wintering and the stopover region.

### Compare human pressure on habitats in both regions

For each species, we defined the study area as the range of a 50 km buffer around the GPS locations of a specific species recorded during the day at their wintering or stopover region (Fig. 1). We chose 50 km because the maximum foraging flight distance for American and European geese are generally smaller than this [8, 16, 17], and hence both used and unused area should be included. We then compared the human pressure on habitats for each species at each region. The 2009 Global Terrestrial Human Footprint Map with a 1 km resolution [13] was used to extract the human pressure on farmland and wetland/grass located within the study area of each bird species in both the wintering and the stopover region. This standardized human footprint map (with a range of 0–50) was generated by integrating remotely sensed and bottom-up survey information that measure direct and indirect human pressures on the environment, including data on the extent of built environments, crop land, pasture land, human population density, night-time lights, roads, railways, and navigable waterways. To test whether human pressure differs between the wintering and stopover region, a Mann-Whitney U test was conducted, as the data were not normally distributed.

### Resource selection function modelling

We tested if geese actively select farmland and wetland/grass close to their roosting area and with a relatively low human pressure, using mixed-effect resource selection function modelling. For each region, within the study area of each species, we used daytime GPS records located on farmland and wetland/grass as presence, and randomly generated an equal number of absences on these two land cover types in the part of the study area where geese were not present (Fig. 1). The minimum distance between an absence point and its closest presence point was set to 1 km. To take the resource availability into account, the number of absences generated on each food resource type was calculated based on the farmland and wetland/grass composition in the study area of each species in each region (tundra bean geese in the Northeast China Plain: 48, 51%; greater white-fronted geese in the Northeast China Plain: 66, 34%; tundra bean geese in the Yangtze River Floodplain: 84, 16%; greater white-fronted geese in the Yangtze River Floodplain: 82, 18%). For each species on each day, the distance to the roost was calculated as the distance from a specific GPS point recorded during the day to the center location of GPS points recorded in the previous night. We then built a mixed-effect resource selection function model [18], estimated by the generalized linear mixed model with a logit link, to predict bird presence for each species at each region separately. Specifically, we used human pressure, food resource (land cover) type (farmland and wetland/grass, with farmland as the baseline), distance to roosts, and their interaction terms as the fixed factors, and bird ID and year as random factors. As we mainly focused on the fixed effects, the Akaike information criterion (AIC) was used to rank the top fixed-effect model first, and random factors were added afterwards. Response curves of factors with a significant effect on bird presence were constructed by fixing all variables other than the variable of interest constant at their median values, and making predictions at regular intervals over the range of the given variable. The response curves of the interaction terms were calculated by fixing all variables other than the two independent factors (1 and 2) in the specific interaction term constant at their median values, and making predictions for factor 1 at regular intervals over the non-outlier range of factor 2. To facilitate comparing among models, we also produced the predictions for factor 1 at a fixed value of factor 2 (e.g., with a fixed distance to the roost). The 95% confidence interval (CI) was calculated using the Wald confidence interval based on the sampling distribution of the Wald statistic in repeated samples. Only the response curve of the interaction term was plotted if both the interaction and the single term were significant. Analyses were carried out in R 3.5.3 [19], using ‘glmmTMB’ and ‘effects’ packages.

## Results

**Fig. 2:**
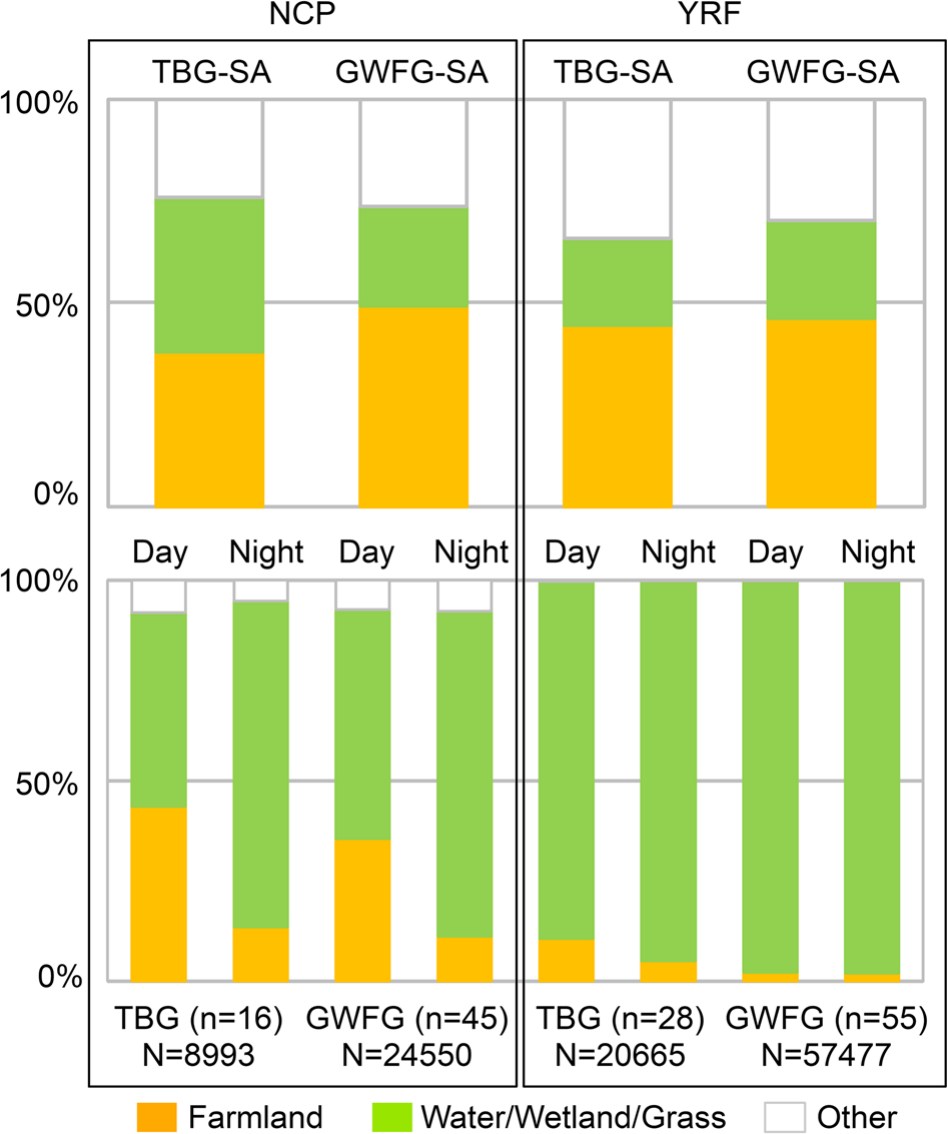
Land cover composition of the study area (above) and habitat use of tracked geese (below). NCP: Northeast China Plain (the stopover region); YRF: Yangtze River Floodplain (the wintering region); TBG tundra bean geese (*Anser serrirostris*); GWFG: greater white-fronted geese (*A. albifrons*); SA: study area (see Fig. 1). Habitat use is quantified by the percentage of GPS locations on specific land cover types. n: number of tracked individuals; N: number of GPS locations

The composition of farmland in the study area of each species in the Northeast China Plain (tundra bean geese = 38%, greater white-fronted geese = 49%) and the Yangtze River Floodplain (tundra bean geese = 45%, greater white-fronted geese = 46%) is similar (Fig. 2). Both goose species mostly stick to their natural habitat in the wintering site Yangtze River Floodplain, with 90% (tundra bean geese) and 98% (greater white-fronted geese) of GPS records during the day located in water/wetland/grass (Fig. 2). In contrast, we show that both goose species do intensively exploit farmland in the stopover site Northeast China Plain, with 44% (tundra bean geese) and 36% (greater white-fronted geese) of GPS records during the day located on farmland (Fig. 2).

**Fig. 3:**
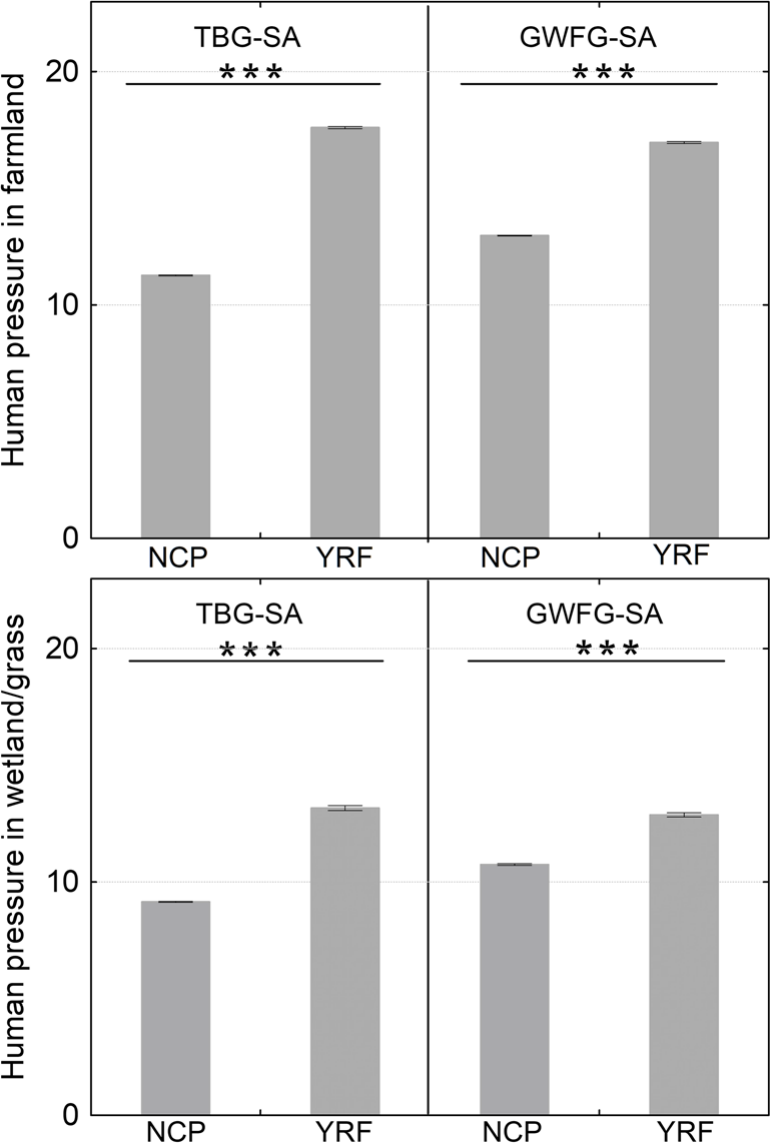
Human pressure on farmland and wetland/grass in the study area. Human pressure refers to the standardized human footprint with a range of 0–50. NCP: Northeast China Plain (the stopover region); YRF: Yangtze River Floodplain (the wintering region); TBG tundra bean geese (*Anser serrirostris*); GWFG: greater white-fronted geese (*A. albifrons*); SA: study area (see Fig. 1). Asterisks indicate a significant difference in human pressure; error bars are 95% confidence intervals around the mean

**Fig. 4:**
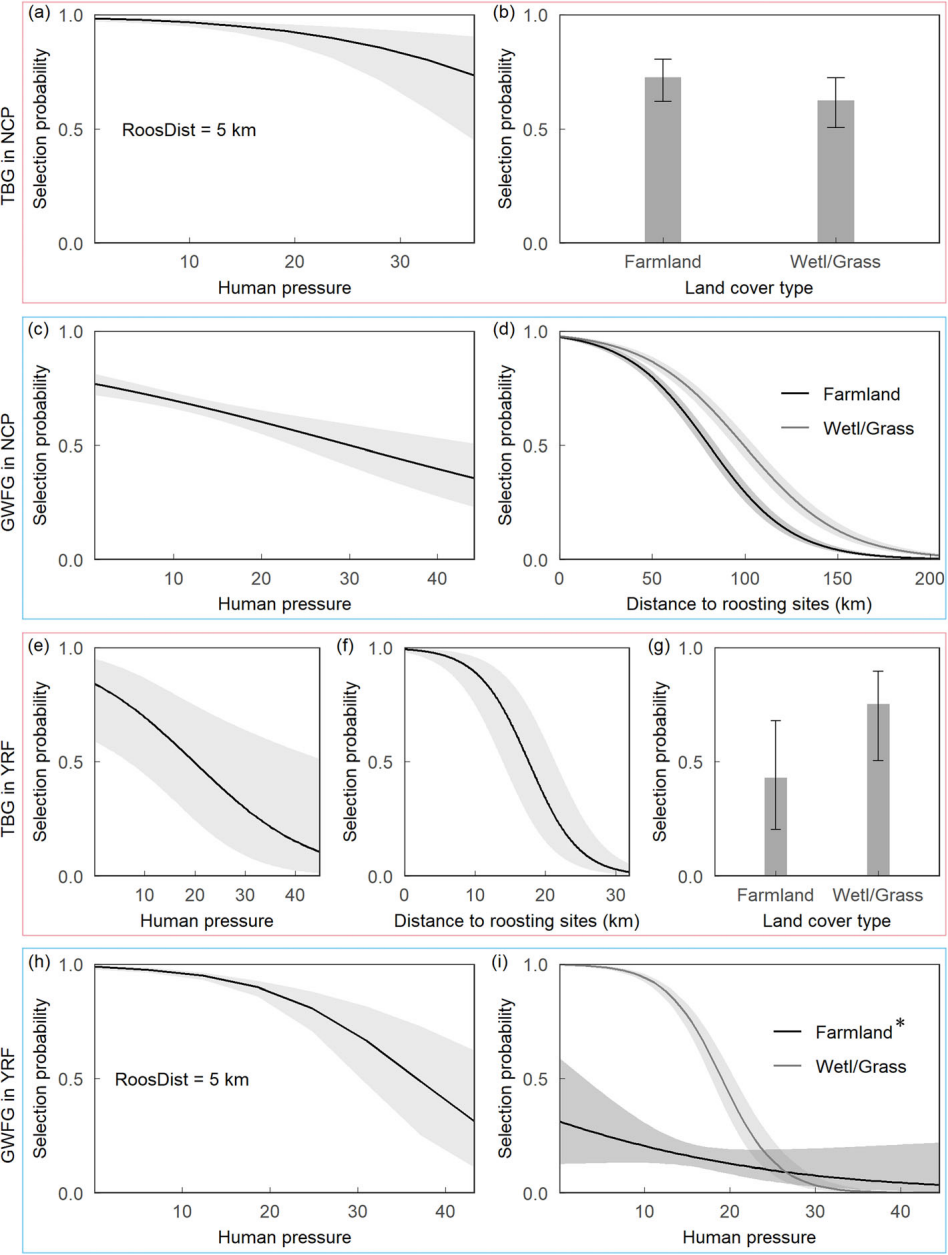
Geese select farmland and wetland/grass near their roosts and experiences relatively low human pressure. Lines indicate response curves, and grey areas and bars 95% confidence intervals. Human pressure refers to the standardized human footprint with a range of 0–50. **a**-**b**: tundra bean geese (*Anser serrirostris*, TBG) in the stopover region Northeast China Plain (NCP); **c**-**d**: greater white-fronted geese (*A. albifrons*, GWFG) in NCP; **e**-**g**: TBG in the wintering region Yangtze River Floodplain (YRF); **h**-**i**: GWFG in YRF. RoostDist: distance to roosting sites; Wetl/Grass: wetland and grass; *: the relatively low selection probability reflects extrapolation beyond the actual level of human pressure (mean = 17.04 and standard deviation = 4.37) based on the relatively little geese presences on farmland

According to the Mann-Whitney U test, human pressure on farmland and wetland/grass in the study area of both goose species is significantly lower in the stopover site Northeast China Plain than in the wintering site Yangtze River Floodplain (Fig. 3; Table 1). We find that roost distance and human pressure show a pronounced effect (individually and/or as an interaction term) on bird presence, suggesting that geese actively select for farmland and/or wetland/grass that is under a lower human pressure and located close to their roosts (Fig. 4a, c, e, f, h, and i; Table 2; Additional file 1: Fig. S1). Both species prefer wetland/grass over farmland, except for tundra bean geese show a higher preference for farmland at the Northeast China Plain (Fig. 4b and g, Table 2).

**Table 1:** Summary of Mann-Whitney U test comparing human pressure level in different regions

Species	Land cover type	Location	Mean	95% CIs	N	U	*p*
TBG	Farmland	YRF	17.593	17.548–17.638	50,278	1.53E+ 10	< 0.001
		NCP	11.286	11.267–11.304	372,100		
	Wetland/grass	YRF	13.174	13.069–13.279	10,456	2.96E+ 9	< 0.001
		NCP	9.146	9.131–9.160	390,058		
GWFG	Farmland	YRF	16.955	16.913–16.998	45,441	1.67E+ 10	< 0.001
		NCP	12.814	12.797–12.831	492,559		
	Wetland/grass	YRF	12.881	12.790–12.972	11,253	1.93E+ 8	< 0.001
		NCP	9.824	9.804–9.845	252,385		

The Northeast China Plain (NPC) stopover site has a significantly lower level of human pressure on the farmland and wetland/grass, in comparison to the Yangtze River Floodplain (YRF) wintering site. Human pressure refers to the standardized human footprint with a range of 0–50. TBG: tundra bean goose (*Anser serrirostris*); GWFG: greater white-fronted goose (*A. albifrons*). CIs: confidence intervals; N: sample size

**Table 2:** Summary of the top resource selection function model quantifying the environmental effect on goose presence

	NCP				YRF			
	TBG		GWFG		TBG		GWFG	
Fixed effects	Estimate	SE	Estimate	SE	Estimate	SE	Estimate	SE
Intercept	−5.36	0.324***	−5.77	0.197***	−14.03	0.828***	−14.78	0.421***
HP	0.35	0.189	−0.21	0.047***	−0.45	0.165**	−3.06	0.643***
RoostDist (km)	−10.83	0.360***	−11.50	0.289***	−22.42	0.937***	−22.43	0.589***
LC	−0.43	0.136**	1.61	0.255***	1.39	0.262***	3.48	0.200***
HP*RoostDist	1.03	0.299***	–	–	–	–	−4.57	0.929***
HP*LC	0.24	0.130	–	–	–	–	−1.54	0.230***
RoostDist*LC	–	–	1.80	0.409***	–	–	–	–
Random effects	Variance	SD	Variance	SD	Variance	SD	Variance	SD
BirdID	0.130	0.361	0.221	0.470	0.977	0.988.	0.390	0.624
Year	0.100	0.317	4.94E-9	7.03E-5	0.848	0.921	0.030	0.172

*NCP* Northeast China Plain, *YRF* Yangtze River Floodplain, *TBG* tundra bean goose (*Anser serrirostris*), *GWFG* greater white-fronted goose (*A. albifrons*), *HP* human pressure (refers to the standardized human footprint with a range of 0–50), *RoostDist* distance to the roost, *LC* land cover type (farmland and wetland/grass, with farmland as the baseline), *BirdID* bird individual ID, *SE* standard error, *SD* standard deviation, *−* no value as the variable is not selected in the best model

***: *P* < 0.001; **: *P* < 0.01; *: *P* < 0.05

## Discussion

We demonstrate that China’s wintering geese can and do venture outside of their threatened natural habitat, in order to exploit the resources offered by farmland, but only under the condition that human pressure is low and the farmland is in close proximity to roosting sites. In the absence of good quality natural habitat during the wintering period, it is critical that geese are able to access the resources provided by farmland, so that sufficient energy can be stored to successfully undertake migration and breeding. Our findings underline that wintering geese seldom exploit farmland in the Yangtze River Floodplain, presumably due to the high level of human pressure on the farmland in this region.

The intensity of human activities is the main factor explaining the difference in use of farmland in the Yangtze River Floodplain wintering site and the Northeast China Plain stopover site. The wintering site is a highly populated area, with human settlements positioned much closer to natural wetlands and surrounding farmlands than in the stopover site. In the stopover site, tundra bean geese prefer farmland over wetland/grass, mainly due to the relatively lower level of human pressure in the farmland, as well as avoiding the competition with greater white-fronted geese, which favor wetland meadows over farmland [20].

Given that habitat loss is identified as the top threat to Chinese wintering waterbirds [21], not being able to explore alternative farmland food resources in the core wintering region likely contributed to the considerable population reduction of Chinese wintering geese. Setting aside farmland refuges, especially at the heavily disturbed key wintering site, the Yangtze River Floodplain, is a promising conservation measure for China’s wintering geese. The Yangtze River Floodplain is one of the most important freshwater ecoregions in the world, containing an exceptional range of biodiversity and environmental conditions [22]. However, this area is also under severe environmental pressure, due to habitat loss, changes in the hydrological regime, pollution and overexploitation [23]. Hence, concrete conservation plans to safeguard this unique ecosystem are particularly urgent. Regarding the Northeast China Plain stopover site, while the current human pressure level allows birds to use farmland to a certain degree, setting farmland refuges would be an effective way to avoid future habitat deterioration.

Allocating part of the total farmland surface as refuges is a tried-and-tested approach that both improves geese survival and reduces goose-agriculture conflicts [10, 11, 24]. Effective design of a refuge network should consider both the selection of suitable farmland patches and improvement of the quality of the designated refuges by management. Furthermore, a compensation scheme for farmers that contribute part of their land to the refuge network needs to be implemented [24].

For the selection of suitable farmland, we suggest using our findings as the primary guideline, to identify suitable farmlands that 1) already experience a relatively low human pressure and 2) are in the close proximity to roosting sites. Furthermore, historic use could be considered to further prioritize suitable farmlands [25]. Food quality and quantity critically influence habitat use [8, 26] and could be used to further improve the selection procedure. In regards to refuge management, measures for minimizing human disturbance and increasing food quality and quantity should be adopted [11]. For the Yangtze River Floodplain wintering site, strict measures should be applied to lower the human disturbance in the designated farmlands, especially during the bird wintering season, such as rechanneling or limiting the use of the intersecting roads, lowering noise pollution (e.g., prohibiting the use of motor bikes and fireworks), and restricting human entry and activities. The food quality and quantity in refuges can be improved further by planting highly nutritious food, or by leaving more grains in field and/or removing free-ranging poultry competing for food. For the Northeast China Plain stopover site, where human pressure is currently relatively low and birds can still use the farmland, measures preventing aggravation of the level of disturbance need to be applied to avoid future deterioration, i.e., changing farmland to built-up area or planning intensive development around the refuges. Lastly, a compensation scheme to farmers would stimulate the implementation of the above measures.

## Conclusions

China’s wintering geese could benefit from farmland if the human pressure on the farmland close to the roosts were to decrease. A network of refuges would open up an as yet practically untapped but abundant source of nutrition for China’s wintering goose populations and could help counter their massive decline – as has already proven effective elsewhere around the world. We recommend that a pilot study is started as soon as possible. If done right, farmland refuges could offer a practical measure for migratory waterfowl conservation in areas of high human-wildlife conflict.

## Supplementary information

**Additional file 1: Figure S1.** Geese select areas experiencing relatively low human pressure, at different distances to roosts. **Table S1.** Summary of GPS records obtained for 83 geese at their stopover and wintering regions.

## Data Availability

The dataset supporting the conclusions of this article is available in the Movebank Data repository (https://www.movebank.org) under ID 52997422, study ‘2015 Tsinghua waterfowl (Yangtze)’. For each individual bird, day and night counts of locations, start and end dates at the wintering and stopover site, and logger information are summarized in Additional file 1: Table S1.

## Abbreviations

GPS: Global Positioning System
GSM: Global System for Mobile Communications
NOAA: The National Oceanic & Atmospheric Administration
AIC: Akaike information criterion
CI: Confidence Interval
