# Supplementary material for: Reducing human pressure on farmland could rescue China’s declining wintering geese
Source: Mov Ecol. 2020 Aug 18;8:35. doi: 10.1186/s40462-020-00220-y (PMC7437007; doi:10.1186/s40462-020-00220-y)
Supplement: Supplementary file 1 — Additional file 1: Figure S1. Geese select areas experiencing relatively low human pressure, at different distances to roosts. Table S1. Summary of GPS records obtained for 83 geese at their stopover and wintering regions. [file 40462_2020_220_MOESM1_ESM.docx]

Additional File 1 - Supplementary figure and table.

Figure S1 Geese select areas experiencing relatively low human pressure, at different distances to roosts.


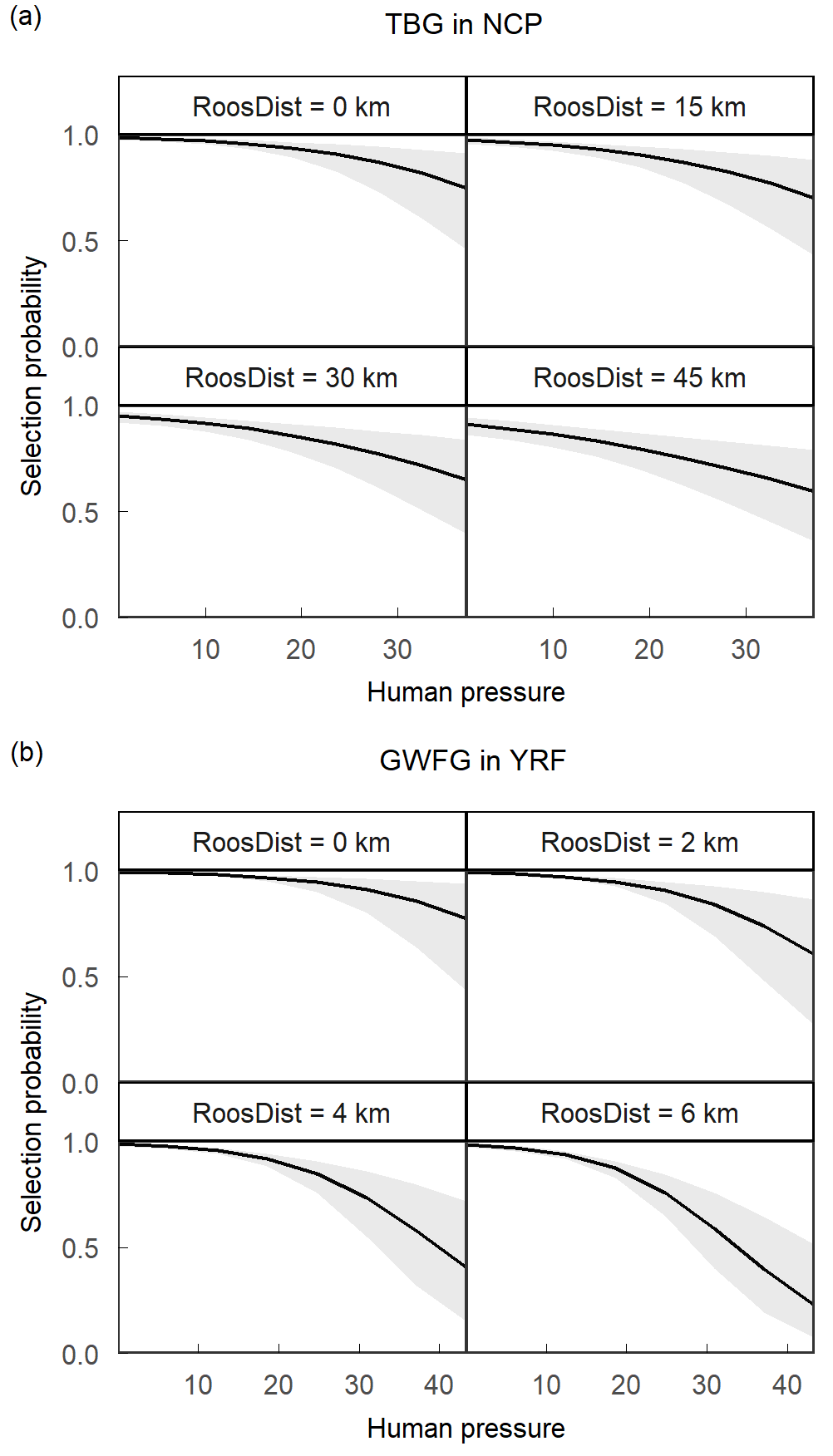


Lines indicate response curves, and grey areas and bars 95% confidence intervals. Human pressure refers to the standardized human footprint with a range of 0-50. (a): tundra bean geese (*Anser serrirostris*, TBG) in the stopover region Northeast China Plain (NCP); (b): greater white-fronted geese (*A. albifrons*, GWFG) in the wintering region Yangtze River Floodplain (YRF); RoostDist: distance to roosts (regular intervals over the non-outlier range).

Table S1 Summary of GPS records obtained for 83 geese at their stopover and wintering regions.

| Species/  Site | Bird ID | Counts of  locations | Day  Counts | Night  Counts | Start Date | End Date | Specie/  Site | Counts of  locations | Day  Counts | Night  Counts | Start Date | End Date |
| --- | --- | --- | --- | --- | --- | --- | --- | --- | --- | --- | --- | --- |
| TBG  (NCP) | E007 | 754 | 399 | 355 | 3/1/2016 | 5/3/2016 | TBG  (YRF) | 825 | 373 | 452 | 12/9/2015 | 2/29/2016 |
|  | E024 | 277 | 152 | 125 | 3/20/2016 | 6/4/2016 |  | 490 | 229 | 261 | 12/12/2015 | 3/7/2016 |
|  | H004 | 454 | 264 | 190 | 3/2/2016 | 5/1/2016 |  | 265 | 121 | 144 | 12/17/2015 | 3/1/2016 |
|  | H019 | 295 | 139 | 156 | 3/2/2016 | 3/26/2016 |  | 556 | 235 | 321 | 12/14/2015 | 3/1/2016 |
|  | H062 | 529 | 311 | 218 | 3/23/2018 | 5/9/2018 |  | 1706 | 789 | 917 | 10/19/2017 | 3/22/2018 |
|  | H090 | 664 | 363 | 301 | 3/4/2018 | 4/30/2018 |  | 853 | 374 | 479 | 11/22/2017 | 2/26/2018 |
|  | H107 | 715 | 387 | 328 | 3/2/2018 | 5/14/2018 |  | 1373 | 581 | 792 | 12/6/2017 | 3/1/2018 |
|  | H118 | 669 | 377 | 292 | 3/10/2018 | 5/14/2018 |  | 1330 | 596 | 734 | 12/7/2017 | 3/9/2018 |
|  | H136 | 754 | 411 | 343 | 3/2/2018 | 5/12/2018 |  | 578 | 262 | 316 | 1/5/2018 | 2/27/2018 |
|  | H137 | 406 | 213 | 193 | 3/10/2018 | 4/21/2018 |  | 677 | 295 | 382 | 1/2/2018 | 3/9/2018 |
|  | H139 | 606 | 329 | 277 | 3/11/2018 | 5/5/2018 |  | 723 | 326 | 397 | 1/1/2018 | 3/10/2018 |
|  | H141 | 645 | 320 | 325 | 2/25/2018 | 4/25/2018 |  | 553 | 236 | 317 | 1/1/2018 | 2/23/2018 |
|  | H145 | 605 | 336 | 269 | 3/12/2018 | 5/11/2018 |  | 921 | 403 | 518 | 1/1/2018 | 3/10/2018 |
|  | K001 | 531 | 275 | 256 | 3/10/2017 | 5/1/2017 |  | 927 | 364 | 563 | 12/5/2016 | 3/9/2017 |
|  | K007 | 540 | 266 | 274 | 3/4/2017 | 4/28/2017 |  | 647 | 267 | 380 | 1/1/2017 | 3/3/2017 |
|  | T0122 | 549 | 289 | 260 | 3/6/2016 | 4/26/2016 |  | 553 | 278 | 275 | 12/15/2015 | 3/5/2016 |
|  | E011 | - | - | - | - | - |  | 555 | 270 | 285 | 12/12/2015 | 2/27/2016 |
|  | E023 | - | - | - | - | - |  | 125 | 57 | 68 | 12/14/2015 | 12/28/2015 |
|  | H022 | - | - | - | - | - |  | 560 | 233 | 327 | 12/14/2015 | 2/26/2016 |
|  | H048 | - | - | - | - | - |  | 118 | 51 | 67 | 12/31/2016 | 1/9/2017 |
|  | H056 | - | - | - | - | - |  | 1286 | 539 | 747 | 10/20/2017 | 3/10/2018 |
|  | H070 | - | - | - | - | - |  | 1581 | 729 | 852 | 11/23/2017 | 4/30/2018 |
|  | H095 | - | - | - | - | - |  | 481 | 192 | 289 | 12/6/2017 | 1/16/2018 |
|  | H122 | - | - | - | - | - |  | 472 | 178 | 294 | 11/22/2017 | 2/17/2018 |
|  | H146 | - | - | - | - | - |  | 1388 | 649 | 739 | 1/1/2018 | 4/9/2018 |
|  | K002 | - | - | - | - | - |  | 669 | 207 | 462 | 12/5/2016 | 2/25/2017 |
|  | K003 | - | - | - | - | - |  | 117 | 54 | 63 | 12/5/2016 | 12/17/2016 |
|  | K006 | - | - | - | - | - |  | 336 | 112 | 224 | 12/31/2016 | 2/6/2017 |
| GWFG  (NCP) | E001 | 453 | 266 | 187 | 3/29/2015 | 5/17/2015 | GWFG  (YRF) | 649 | 383 | 266 | 1/31/2015 | 3/28/2015 |
|  | E002 | 580 | 342 | 238 | 3/27/2017 | 5/15/2017 |  | 1619 | 741 | 878 | 10/10/2015 | 3/25/2016 |
|  | E003 | 561 | 326 | 235 | 3/27/2015 | 5/21/2015 |  | 952 | 435 | 517 | 11/10/2015 | 3/26/2016 |
|  | E005 | 494 | 288 | 206 | 3/23/2015 | 5/14/2015 |  | 843 | 395 | 448 | 10/31/2015 | 3/25/2016 |
|  | E010 | 651 | 381 | 270 | 3/28/2017 | 5/22/2017 |  | 1202 | 574 | 628 | 10/13/2016 | 3/27/2017 |
|  | E013 | 575 | 341 | 234 | 3/23/2018 | 5/17/2018 |  | 949 | 444 | 505 | 10/23/2017 | 3/22/2018 |
|  | E017 | 505 | 301 | 204 | 3/27/2016 | 5/8/2016 |  | 767 | 342 | 425 | 12/13/2015 | 3/25/2016 |
|  | E018 | 487 | 282 | 205 | 3/28/2016 | 5/8/2016 |  | 1051 | 500 | 551 | 12/13/2015 | 3/27/2016 |
|  | E022 | 443 | 255 | 188 | 3/29/2017 | 5/16/2017 |  | 1063 | 499 | 564 | 10/29/2016 | 10/20/2017 |
|  | H003 | 499 | 302 | 197 | 3/28/2016 | 5/10/2016 |  | 999 | 450 | 549 | 12/14/2015 | 3/27/2016 |
|  | H005 | 395 | 232 | 163 | 3/28/2016 | 5/9/2016 |  | 867 | 382 | 485 | 12/14/2015 | 3/27/2016 |
|  | H006 | 429 | 253 | 176 | 3/28/2016 | 5/9/2016 |  | 784 | 354 | 430 | 12/14/2015 | 3/28/2016 |
|  | H016 | 509 | 310 | 199 | 3/28/2016 | 5/19/2016 |  | 818 | 361 | 457 | 12/14/2015 | 3/25/2016 |
|  | H018 | 288 | 175 | 113 | 4/20/2016 | 5/17/2016 |  | 963 | 437 | 526 | 12/14/2015 | 4/18/2016 |
|  | H021 | 503 | 293 | 210 | 3/27/2016 | 5/9/2016 |  | 880 | 399 | 481 | 12/14/2015 | 3/26/2016 |
|  | H050 | 1190 | 697 | 493 | 3/28/2017 | 5/16/2017 |  | 959 | 401 | 558 | 12/7/2016 | 3/26/2017 |
|  | H057 | 601 | 357 | 244 | 3/24/2018 | 5/14/2018 |  | 2075 | 927 | 1148 | 10/19/2017 | 3/23/2018 |
|  | H063 | 626 | 367 | 259 | 3/23/2018 | 5/14/2018 |  | 1639 | 731 | 908 | 10/19/2017 | 3/22/2018 |
|  | H064 | 511 | 298 | 213 | 3/27/2018 | 5/14/2018 |  | 829 | 373 | 456 | 1/1/2018 | 3/27/2018 |
|  | H066 | 1173 | 681 | 492 | 3/23/2018 | 5/12/2018 |  | 2295 | 1037 | 1258 | 10/19/2017 | 3/22/2018 |
|  | H067 | 506 | 307 | 199 | 3/24/2018 | 5/10/2018 |  | 1111 | 475 | 636 | 11/22/2017 | 3/23/2018 |
|  | H071 | 599 | 352 | 247 | 3/24/2018 | 5/14/2018 |  | 1224 | 533 | 691 | 11/16/2017 | 3/23/2018 |
|  | H072 | 613 | 359 | 254 | 3/23/2018 | 5/14/2018 |  | 1081 | 445 | 636 | 11/17/2017 | 3/22/2018 |
|  | H074 | 357 | 204 | 153 | 3/22/2018 | 4/24/2018 |  | 954 | 376 | 578 | 11/26/2017 | 3/21/2018 |
|  | H079 | 733 | 425 | 308 | 3/22/2018 | 5/2/2018 |  | 1073 | 453 | 620 | 11/21/2017 | 3/21/2018 |
|  | H081 | 126 | 75 | 51 | 3/27/2018 | 4/7/2018 |  | 1170 | 526 | 644 | 11/20/2017 | 3/26/2018 |
|  | H086 | 622 | 364 | 258 | 3/23/2018 | 5/14/2018 |  | 1027 | 441 | 586 | 11/22/2017 | 3/22/2018 |
|  | H088 | 511 | 304 | 207 | 3/23/2018 | 5/7/2018 |  | 1335 | 579 | 756 | 11/16/2017 | 3/22/2018 |
|  | H089 | 296 | 169 | 127 | 3/24/2018 | 4/18/2018 |  | 1080 | 450 | 630 | 11/22/2017 | 3/23/2018 |
|  | H092 | 504 | 294 | 210 | 3/29/2018 | 5/14/2018 |  | 1244 | 545 | 699 | 11/22/2017 | 3/27/2018 |
|  | H093 | 560 | 326 | 234 | 3/28/2018 | 5/14/2018 |  | 1057 | 451 | 606 | 12/3/2017 | 3/27/2018 |
|  | H094 | 543 | 317 | 226 | 3/29/2018 | 5/14/2018 |  | 1158 | 499 | 659 | 11/18/2017 | 3/28/2018 |
|  | H097 | 409 | 233 | 176 | 4/7/2018 | 5/14/2018 |  | 1315 | 569 | 746 | 11/18/2017 | 3/27/2018 |
|  | H100 | 565 | 327 | 238 | 3/27/2018 | 5/14/2018 |  | 1138 | 489 | 649 | 11/16/2017 | 3/27/2018 |
|  | H115 | 593 | 346 | 247 | 3/24/2018 | 5/14/2018 |  | 1026 | 421 | 605 | 11/19/2017 | 3/23/2018 |
|  | H124 | 507 | 292 | 215 | 3/25/2018 | 5/10/2018 |  | 656 | 266 | 390 | 12/3/2017 | 3/23/2018 |
|  | H125 | 589 | 345 | 244 | 3/25/2018 | 5/14/2018 |  | 1199 | 527 | 672 | 11/18/2017 | 3/24/2018 |
|  | H127 | 600 | 354 | 246 | 3/18/2018 | 5/14/2018 |  | 1410 | 618 | 792 | 11/16/2017 | 3/24/2018 |
|  | H128 | 614 | 354 | 260 | 3/22/2018 | 5/14/2018 |  | 1230 | 515 | 715 | 11/18/2017 | 3/22/2018 |
|  | H129 | 524 | 296 | 228 | 3/27/2018 | 5/12/2018 |  | 1366 | 590 | 776 | 11/18/2017 | 3/27/2018 |
|  | H131 | 593 | 347 | 246 | 3/23/2018 | 5/14/2018 |  | 1137 | 484 | 653 | 11/20/2017 | 3/22/2018 |
|  | H133 | 137 | 67 | 70 | 3/28/2018 | 5/13/2018 |  | 490 | 191 | 299 | 11/18/2017 | 3/27/2018 |
|  | H134 | 404 | 217 | 187 | 3/23/2018 | 5/1/2018 |  | 840 | 367 | 473 | 1/2/2018 | 3/22/2018 |
|  | H140 | 1062 | 606 | 456 | 3/27/2018 | 5/13/2018 |  | 1399 | 652 | 747 | 1/1/2018 | 3/27/2018 |
|  | H144 | 510 | 298 | 212 | 3/27/2018 | 5/9/2018 |  | 1073 | 482 | 591 | 1/1/2018 | 3/26/2018 |
|  | E015 | - | - | - | - | - |  | 134 | 58 | 76 | 12/13/2015 | 12/28/2015 |
|  | E021 | - | - | - | - | - |  | 205 | 95 | 110 | 12/11/2015 | 1/6/2016 |
|  | H013 | - | - | - | - | - |  | 327 | 130 | 197 | 12/14/2015 | 2/4/2016 |
|  | H047 | - | - | - | - | - |  | 1388 | 613 | 775 | 12/5/2016 | 4/7/2017 |
|  | H061 | - | - | - | - | - |  | 639 | 302 | 337 | 10/19/2017 | 12/1/2017 |
|  | H065 | - | - | - | - | - |  | 716 | 326 | 390 | 10/19/2017 | 11/20/2017 |
|  | H117 | - | - | - | - | - |  | 498 | 213 | 285 | 11/16/2017 | 4/11/2018 |
|  | H121 | - | - | - | - | - |  | 1458 | 654 | 804 | 11/17/2017 | 3/26/2018 |
|  | H142 | - | - | - | - | - |  | 1048 | 467 | 581 | 1/1/2018 | 3/26/2018 |
|  | H143 | - | - | - | - | - |  | 1093 | 493 | 600 | 1/1/2018 | 4/8/2018 |

TBG: Tundra Bean Geese (*Anser serrirostris*); GWFG: Greater White-fronted Geese (*A. albifrons*); NCP: Northeast China Plain stopover region; YRF: the Yangtze River Floodplain wintering region; Bird ID: E - 22g neck band (< 1% of body weight) from Ecotone telemetry, Gdynia, Poland; H - 26 g neck band (< 1% of body weight) from Hunan Global Messenger Technology Co. Ltd, Xiangtan, China; K - 72g neck band (< 3% of body weight) from KoEco Inc., South Korea; T - 25g backpack logger (< 1% of body weight) from Blueoceanix Technology Co. Ltd, Tianjin, China.
